# Supplementary figures and images for: Evaluation of therapeutic agent selection based on comprehensive genomic profiling in gastroenteropancreatic neuroendocrine neoplasms
Source: PLoS One. 2025 Aug 6;20(8):e0325727. doi: 10.1371/journal.pone.0325727 (PMC12327669; doi:10.1371/journal.pone.0325727)

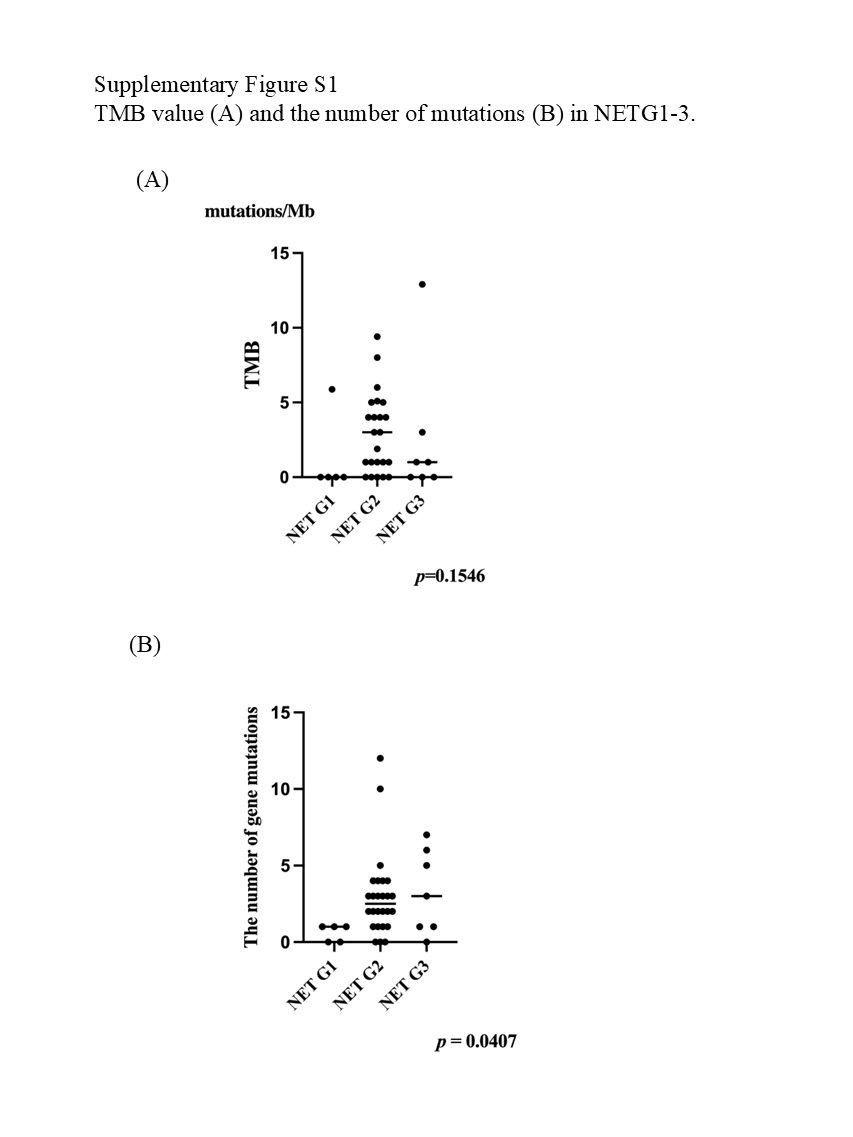

Supplement: S1 Fig — (TIF) [file pone.0325727.s001.tif]

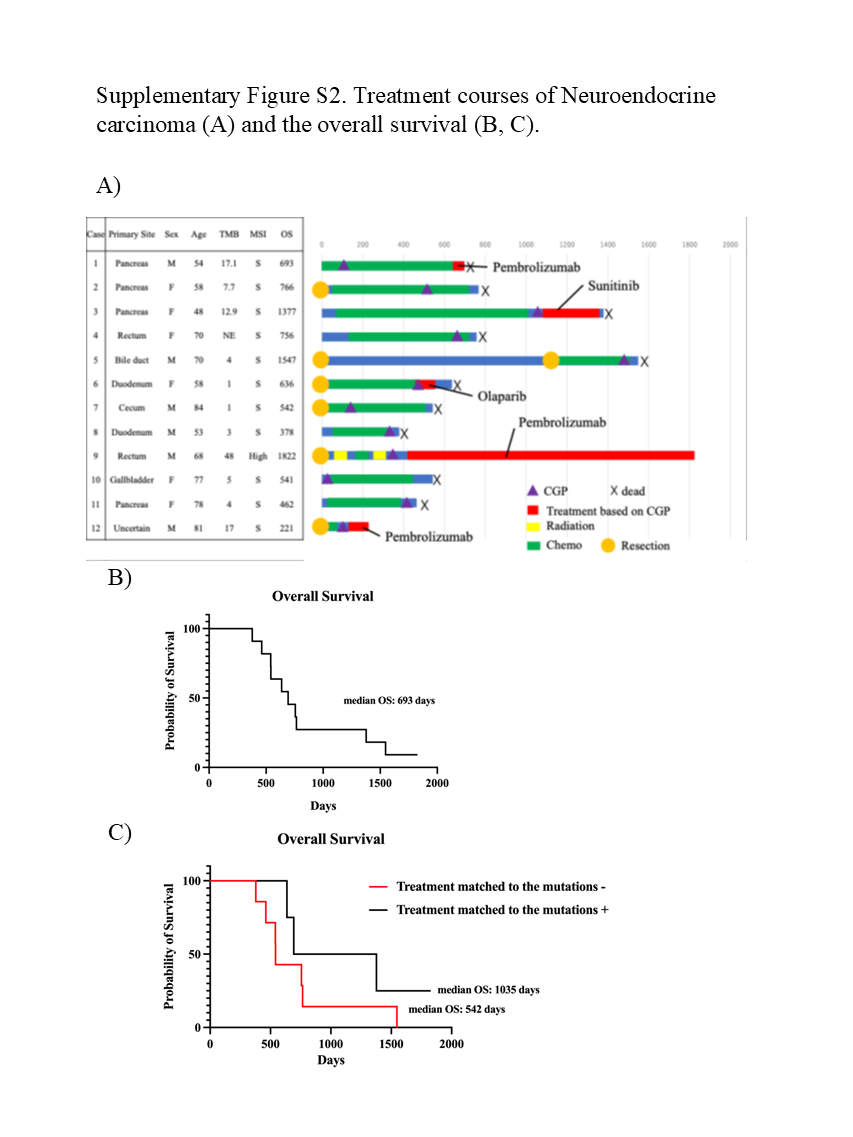

Supplement: S2 Fig — Kaplan–Meier curves depicting overall survival. (B, C). (TIF) [file pone.0325727.s002.tif]
