# Supplementary material for: Evaluation of therapeutic agent selection based on comprehensive genomic profiling in gastroenteropancreatic neuroendocrine neoplasms
Source: PLoS One. 2025 Aug 6;20(8):e0325727. doi: 10.1371/journal.pone.0325727 (PMC12327669; doi:10.1371/journal.pone.0325727)
Supplement: Attachment — Submitted filename: plos one.pdf [file pone.0325727.s004.pdf]

Revision of the manuscript entitled:

"Evaluation of therapeutic agent selection based on comprehensive genomic profiling in gastrointestinal pancreatic neuroendocrine neoplasms"

Authors: Suguru Miyazawa, Hiroaki Ono, Hironari Yamashita, Daisuke Asano, Yoshiya Ishikawa, Shuichi Watanabe Hiroki Ueda, Satoru Aoyama, Naoya Ishibashi, Keiichi Akahoshi, Sadakatsu Ikeda, Minoru Tanabe

This article aims to provide a comprehensive insight into the evaluation of therapeutic agents for gastrointestinal pancreatic neuroendocrine neoplasms based on genomic profiling. While the topic is indeed interesting and holds potential for publication, several aspects require significant improvement:

1. Please ensure that all abbreviations are fully spelled out upon their first mention in the manuscript. This will enhance clarity, particularly for readers who may not be familiar with these terms.
2. Many of the abbreviations used (e.g., MiNEN, GEP-NENs, NEC, NET, NEN) are complex and quite similar, which may lead to confusion. Consider revising the choice of abbreviations to improve readability and comprehension.
3. Titles and subtitles consisting solely of abbreviations, such as the one in line 104, are uncommon. I suggest reconsidering this approach by either spelling out the abbreviation or providing a more descriptive heading to enhance readability.
4. The text would benefit from significant revision to improve fluidity and overall readability. The frequent use of abbreviations, particularly those that are similar in form, makes comprehension more challenging. Reducing their frequency or restructuring certain sections could help create a smoother reading experience.
5. The methodology section requires further elaboration. Please provide more details regarding the processing of tissue and biopsy samples to ensure methodological transparency.
6. The role of the Molecular Tumor Board should be further clarified. How were opinions aligned, and what was the decision-making process regarding treatment options? A more detailed explanation of their involvement would strengthen the manuscript.

By addressing these points, the paper's clarity, readability, and scientific rigor will be significantly enhanced.
